# Development of a novel nomogram-based online tool to predict axillary status after neoadjuvant chemotherapy in cN+ breast cancer: A multicentre study on 1,950 patients

**DOI:** 10.1016/j.breast.2021.09.013

**Published:** 2021-10-02

**Authors:** Fabio Corsi, Sara Albasini, Luca Sorrentino, Giulia Armatura, Claudia Carolla, Corrado Chiappa, Francesca Combi, Annalisa Curcio, Angelica Della Valle, Guglielmo Ferrari, Maria Luisa Gasparri, Oreste Gentilini, Matteo Ghilli, Chiara Listorti, Stefano Mancini, Peter Marinello, Francesco Meani, Simone Mele, Anna Pertusati, Manuela Roncella, Francesca Rovera, Adele Sgarella, Giovanni Tazzioli, Daniela Tognali, Secondo Folli

**Affiliations:** a Breast Unit, Department of Surgery, Istituti Clinici Scientifici Maugeri IRCCS, Pavia, Italy; b Department of Biomedical and Clinical Sciences “Luigi Sacco”, Università di Milano, Milan, Italy; c Chirurgia Generale, Ospedale Centrale di Bolzano, Azienda Sanitaria dell'Alto Adige, Italy; d Breast Unit, Surgery, Fondazione IRCCS Istituto Nazionale dei Tumori, Milan, Italy; e SSD Breast Unit, ASST-Settelaghi di Varese, Italy; f Breast Unit Azienda Ospedaliero-Universitaria Policlinico Modena, Italy; g Chirurgia Senologica, Ospedale Morgagni Pierantoni, Ausl Romagna, Forlì, Italy; h Breast Surgery, Department of Surgery, Fondazione IRCCS Policlinico San Matteo, Pavia, Italy; i Breast Surgery Unit, AUSL-IRCCS Reggio Emilia, Via Amendola 2, 42122, Reggio Emilia, Italy; j Service of Gynecology and Obstetrics, Department of Gynecology and Obstetrics, Ospedale Regionale di Lugano EOC, Lugano, Switzerland; k Faculty of Biomedical Sciences, Università della Svizzera Italiana, Lugano, Switzerland; l Breast Surgery, San Raffaele University and Research Hospital, Milano, Italy; m Breast Cancer Centre, University Hospital of Pisa, Italy; n Breast Surgery, Department of Surgery, ASST Fatebenefratelli Sacco, Milano, Italy; o General Surgery I, Department of Surgery, ASST Fatebenefratelli Sacco, Milano, Italy; p Università degli Studi di Pavia, Pavia, Italy

**Keywords:** Breast cancer, Neoadjuvant chemotherapy, Axillary surgery, Axillary dissection, Sentinel node biopsy

## Abstract

**Background:**

Type of axillary surgery in breast cancer (BC) patients who convert from cN + to ycN0 after neoadjuvant chemotherapy (NAC) is still debated. The aim of the present study was to develop and validate a preoperative predictive nomogram to select those patients with a low risk of residual axillary disease after NAC, in whom axillary surgery could be minimized.

**Patients and methods:**

1950 clinically node-positive BC patients from 11 Breast Units, treated by NAC and subsequent surgery, were included from 2005 to 2020. Patients were divided in two groups: those who achieved nodal pCR vs. those with residual nodal disease after NAC. The cohort was divided into training and validation set with a geographic separation criterion. The outcome was to identify independent predictors of axillary pathologic complete response (pCR).

**Results:**

Independent predictive factors associated to nodal pCR were axillary clinical complete response (cCR) after NAC (OR 3.11, p < 0.0001), ER-/HER2+ (OR 3.26, p < 0.0001) or ER+/HER2+ (OR 2.26, p = 0.0002) or ER-/HER2- (OR 1.89, p = 0.009) BC, breast cCR (OR 2.48, p < 0.0001), Ki67 > 14% (OR 0.52, p = 0.0005), and tumor grading G2 (OR 0.35, p = 0.002) or G3 (OR 0.29, p = 0.0003). The nomogram showed a sensitivity of 71% and a specificity of 73% (AUC 0.77, 95%CI 0.75–0.80). After external validation the accuracy of the nomogram was confirmed.

**Conclusion:**

The accuracy makes this freely-available, nomogram-based online tool useful to predict nodal pCR after NAC, translating the concept of tailored axillary surgery also in this setting of patients.

## Highlights

• Axillary surgery after neoadjuvant chemotherapy in breast cancer is still debated.
• Several surgical approaches are reported but clear evidences are lacking.
• Prediction of axillary status after neoadjuvant chemotherapy is a clinical need.
• An nomogram was developed and validated to preoperatively identify ypN0 patients.
• This online tool could allow de-escalation of axillary surgery.

## 1 Introduction

Neoadjuvant chemotherapy (NAC) is increasingly used in the treatment of breast cancer [1,2]. The wide adoption of NAC has led to 1) an increase in breast-conserving and axillary-saving surgery and 2) useful information on patients’ responsiveness to chemotherapy, also considering that response to NAC might predict survival outcomes [[3], [4], [5]]. However, axillary management after NAC is a controversial matter of debate [6]. In breast cancer patients with axillary involvement at presentation, NAC may downstage axillary disease [7]. Until a few years ago, axillary lymph node dissection (ALND) has been the standard of care, irrespectively of nodal status after completion of NAC [8]. However, nodal pathologic complete response (pCR) is well documented in about 40–70% of women presenting with cN + status at baseline [[9], [10], [11]]. In this subset of patients, upfront ALND could be therefore unnecessary. First landmark trials have assessed the feasibility of sentinel lymph node (SLN) biopsy also for patients who had clinical conversion from cN + to ycN0, if at least 3 SLNs are retrieved [12,13]. Moreover, recent evidences suggested no difference in 10-yr disease-free survival rates if these patients are treated by SLN biopsy only [14,15]. Different approaches are currently accepted, including sampling of lymph nodes, SLN biopsy technique with double tracer, targeted axillary dissection (TAD) or ALND [[16], [17], [18]]. Furthermore, while ALND could be avoided in case of 1–2 metastatic SLNs in primary surgery setting, the oncologic safety of this approach after NAC is an open field of research [19,20]. A predictive model to assess the individual likelihood of axillary pCR after NAC, could help to select those patients who might be suitable for mini-invasive axillary surgery, minimizing the need of ALND even in case of residual nodal disease in the SLN. The aim of the present study is to develop and validate a nomogram-based online tool, to be used in routine clinical practice, to estimate the ypN0 probability of each patient.

## 2 Materials and methods

### 2.1 Study population

A multicentre retrospective study was performed including clinically node-positive breast cancer patients treated by NAC and subsequent surgery from 2005 to 2020 in 11 tertiary breast units. The study was promoted and coordinated by the Breast Unit of IRCCS Maugeri Hospital of Pavia, Italy. Inclusion criteria were: a biopsy-proven diagnosis of breast cancer, node-positive disease evaluated clinically and by imaging (included ultrasound and/or magnetic resonance imaging [MRI] and/or positron emission tomography [PET]), with or without a fine-needle aspiration cytology (FNAC) or core biopsy, administration of NAC, any axillary surgery after NAC, and clinical/radiological re-assessment of the axilla with any type of imaging, according to clinical practice of each breast unit. Exclusion criteria were: contraindicated chemotherapy and distant metastases at diagnosis. The study protocol was approved by the Ethical Committee of the coordinating institution (protocol LN-NEO 01, approval number 2394CE) and by the ethical committees of all the participating centres. Data were collected in a GDPR-compliant ad-hoc database accessed only by the study investigators.

### 2.2 Evaluation of clinical and pathological response

After NAC completion within 1–4 weeks before surgery, all patients were re-staged by clinical evaluation, digital mammography, breast/axillary ultrasound and/or MRI and/or PET to evaluate clinical response to NAC. Re-staging was performed in each breast unit with the same imaging techniques used in the initial staging before NAC. RECIST criteria (version 1.1) were applied to define the breast clinical response [21,22]. Specifically, clinical complete response (cCR) was defined as no residual tumor nor microcalcifications visible on post-NAC imaging, being all the target lesions disappeared. After surgery, breast pCR was defined as the absence of residual invasive cancer on final pathology (ypT0/Tis in the current AJCC staging system) [23]. Axillary clinical response was evaluated based on the presence or absence of abnormal/enlarged lymph nodes (focally or diffusely >3 mm thickened cortex, deformed/absent fatty hilum). Based on axillary clinical response, patients were treated either by ALND or SLN biopsy. In the latter case, identification of SLNs was performed either by single or double tracer, using Tc99 radioisotope or indocyanine green or blue dye depending on the Breast Unit; at least 2–3 SLNs were excised for each patient. If isolated tumor cells, micro- or macrometastases were identified in the SLNs, a complete axillary dissection was performed. Axillary pCR was defined as no micro- or macrometastases in any excised lymph node (ypN0/ITC+).

#### 2.2.1 Endpoints and study design

The primary endpoint was to identify independent predictors of axillary pCR after NAC, developing and externally validating a dedicated nomogram. For this purpose, patients were divided in two groups: those who achieved nodal pCR vs. those with residual nodal disease after NAC. The cohort was divided into two different subsets: the training set was composed by patients from 8 centres and used to develop the nomogram, while the validation set was composed by the remaining 3 centres and it was used to externally validate the nomogram. A geographic external validation was chosen [[24], [25], [26]]. The analyses used to develop and validate the nomogram are described in Supplementary Methods.

An easy-to-use, freely-available, nomogram-based online tool (LNNeo) to predict the axillary status after NAC has been developed (https://app.linfoneo.com/).

### 2.3 Statistical analyses

Variables were reported as means ± standard deviations or as absolute numbers and percentages. Statistical significance was set at p < 0.05 (two tailed). Data analysis was performed using SAS software (v. 9.4, SAS Institute Inc., Cary, USA) and R software (v. 3.5.1, © The R Foundation); see Supplementary Methods for details.

## 3 Results

### 3.1 Baseline characteristics of study population

A total of 1950 cN + breast cancer patients treated by NAC were included in the study. In 1389 patients (71.2%) the initial staging of axilla was performed by ultrasound, while in 559 patients (28.7%) MRI and/or PET was preferred, and in 2 patients (0.1%) a computerized tomography was used. In 641 patients (32.9%) an axillary FNAC or core biopsy was used to confirm nodal involvement before NAC. In 886 cases (45.4%) axillary pCR was observed on final pathology, while in 1064 patients (54.6%) residual nodal disease was found after surgery. The training set included 1447 patients (74.2%), while the validation set included 503 patients (25.8%). Initial clinical nodal staging was cN1 in 85.8%, cN2 in 10.6%, and cN3 in 3.6% of cases. All the baseline variables between the two groups are reported in Table 1.

**Table 1:** Baseline features between patients with or without axillary pCR.

	Total patients			Training set			External validation set		
	Axillary pCR (n = 886)	No axillary pCR (n = 1064)	P Value	Axillary pCR (n = 645)	No axillary pCR (n = 802)	P Value	Axillary pCR (n = 241)	No axillary pCR (n = 262)	P Value
**Age at diagnosis (years)**	51 ± 12 [25–91]	53 ± 12 [25–89]	0.008	51 ± 12 [25–91]	53 ± 12 [25–87]	0.02	51 ± 11 [26–84]	52 ± 12 [25–89]	0.27
**Body Mass Index (BMI)**	25.1 ± 5.1 [13.1–47.3]	25.4 ± 4.7 [15.2–52.0]	0.13	25.1 ± 5.0 [14.9–47.3]	25.4 ± 4.6 [15.2–43.8]	0.14	25.2 ± 5.4 [13.1–42.6]	25.3 ± 5.0 [16.6–52.0]	0.59
**Lesion size on imaging (mm)**	37.5 ± 19.1 [4.0–130.0]	38.5 ± 19.7 [6.0–170.0]	0.08	39.2 ± 20.0 [4.0–130.0]	39.4 ± 19.9 [6.0–170.0]	0.73	33.0 ± 15.7 [9.0–97.0]	37.4 ± 19.1 [6.0–150.0]	0.009
**Axillary node size on imaging (mm)**	18.7 ± 7.9 [5.0–58.0]	21.0 ± 10.6 [2.0–100.0]	0.0003	18.3 ± 7.4 [5.0–58.0]	20.6 ± 10.2 [2.0–75.0]	0.002	19.6 ± 9.1 [6.0–50.0]	22.0 ± 11.4 [6.0–100.0]	0.05
**Multifocal disease on imaging**									
No	668 (75.5%)	787 (74.1%)	0.50	509 (79.0%)	616 (76.9%)	0.34	159 (66.0%)	171 (65.5%)	0.92
Yes	217 (24.5%)	275 (25.9%)		135 (21.0%)	185 (23.1%)		82 (34.0%)	90 (34.5%)	
**Pre-treatment clinical T stage**									
cT1	126 (14.3%)	117 (11.0%)	0.03	86 (13.4%)	95 (11.9%)	0.42	40 (16.6%)	22 (8.4%)	0.01
cT2	502 (56.8%)	586 (55.3%)		364 (56.8%)	443 (55.4%)		138 (57.3%)	143 (54.6%)	
cT3	131 (14.9%)	172 (16.2%)		99 (15.4%)	122 (15.3%)		32 (13.3%)	50 (19.1%)	
cT4	123 (14.0%)	186 (17.5%)		92 (14.4%)	139 (17.4%)		31 (12.8%)	47 (17.9%)	
**Pre-treatment clinical N stage**									
cN1	793 (89.5%)	880 (82.7%)	<0.0001	565 (87.6.0%)	648 (80.8%)	0.002	228 (94.6%)	232 (88.5%)	0.02
cN2	70 (7.9%)	137 (12.9%)		58 (9.0%)	115 (14.3%)		12 (5.0%)	22 (8.4%)	
cN3	23 (2.6%)	47 (4.4%)		22 (3.5%)	39 (4.9%)		1 (0.4%)	8 (3.1%)	
**Histological type**^a^									
Invasive ductal	695 (78.6%)	792 (74.5%)	<0.0001	497 (77.2%)	598 (74.6%)	0.002	198 (82.2%)	194 (74.3%)	0.0008
Invasive lobular	34 (3.8%)	100 (9.4%)		27 (4.2%)	70 (8.7%)		7 (2.9%)	30 (11.5%)	
Others	156 (17.6%)	171 (16.1%)		120 (18.6%)	134 (16.7%)		36 (14.9%)	37 (14.2%)	
**Grading**^a^									
G1	35 (4.3%)	21 (2.1%)	<0.0001	32 (5.2%)	18 (2.4%)	<0.0001	3 (1.4%)	3 (1.4%)	<0.0001
G2	256 (31.1%)	481 (48.5%)		198 (32.4%)	369 (48.2%)		58 (27.5%)	112 (49.3%)	
G3	531 (64.6%)	490 (49.4%)		381 (62.4%)	378 (49.4%)		150 (71.1%)	112 (49.3%)	
**Biomolecular subtype**^a^									
ER+/Her2-	239 (27.1%)	634 (60.2%)	<0.0001	179 (27.9%)	466 (58.6%)	<0.0001	60 (24.8%)	168 (64.9%)	<0.0001
ER+/Her2+	236 (26.8%)	167 (15.8%)		179 (27.9%)	130 (16.4%)		57 (23.7%)	37 (14.3%)	
ER-/Her2+	198 (22.4%)	90 (8.6%)		147 (22.9%)	79 (10.0%)		51 (21.2%)	11 (4.2%)	
ER-/Her2-	209 (23.7%)	162 (15.4%)		136 (21.3%)	119 (15.0%)		73 (30.3%)	43 (16.6%)	
**Progesterone receptors**^a^									
Negative	489 (55.2%)	363 (34.1%)	<0.0001	344 (53.3%)	285 (35.5%)	<0.0001	145 (60.2%)	78 (29.8%)	<0.0001
Positive	397 (44.8%)	701 (65.9%)		301 (46.7%)	517 (64.5%)		96 (39.8%)	184 (70.2%)	
**Ki67 index**^a^									
≤14%	67 (7.6%)	201 (19.0%)	<0.0001	57 (8.9%)	160 (20.1%)	<0.0001	10 (4.2%)	41 (15.7%)	<0.0001
>14%	813 (92.4%)	855 (81.0%)		583 (91.1%)	635 (79.9%)		230 (95.8%)	220 (84.3%)	
**Neoadjuvant chemotherapy**									
Anthracyclines/FEC (Type 1)	80 (9.1%)	169 (16.0%)	<0.0001	74 (11.6%)	159 (20.0%)	<0.0001	6 (2.5%)	10 (3.8%)	0.0002
Anthracyclines/FEC + Taxanes (Type 2)	326 (37.1%)	556 (52.5%)		241 (37.8%)	432 (54.3%)		85 (35.3%)	124 (47.3%)	
Anthracyclines/FEC + Taxanes + anti-HER2 (Type 3)	359 (40.9%)	195 (18.5%)		282 (44.3%)	154 (19.4%)		77 (32.0%)	41 (15.7%)	
Others (Type 4)	113 (12.9%)	137 (13.0%)		40 (6.3%)	50 (6.3%)		73 (30.2%)	87 (33.2%)	
**Post-NAC breast cCR**									
No	487 (55.0%)	901 (84.7%)	<0.0001	358 (55.5%)	689 (85.9%)	<0.0001	129 (53.5%)	212 (80.9%)	<0.0001
Yes	399 (45.0%)	163 (15.3%)		287 (44.5%)	113 (14.1%)		112 (46.5%)	50 (19.1%)	
**Post-NAC clinical axillary status**									
Negative	543 (61.6%)	340 (32.4%)	<0.0001	361 (56.3%)	226 (28.6%)	<0.0001	182 (75.8%)	114 (43.5%)	<0.0001
Positive	338 (38.4%)	711 (67.6%)		280 (43.7%)	563 (71.4%)		58 (24.2%)	148 (56.5%)	
**Type of breast surgery**									
Breast-conserving surgery	360 (40.7%)	388 (36.5%)	0.06	267 (41.5%)	319 (39.8%)	0.52	93 (38.6%)	69 (26.3%)	0.004
Total mastectomy	525 (59.3%)	676 (63.5%)		377 (58.5%)	483 (60.2%)		148 (61.4%)	193 (73.7%)	
**Type of axillary surgery**									
SLN biopsy	204 (23.0%)	19 (1.8%)	<0.0001	76 (11.8%)	5 (0.6%)	<0.0001	128 (53.1%)	14 (5.3%)	<0.0001
Axillary dissection	681 (77.0%)	1045 (98.2%)		568 (88.2%)	797 (99.4%)		113 (46.9%)	248 (94.7%)	
**Breast pCR**									
No	363 (41.0%)	937 (88.1%)	<0.0001	267 (41.4%)	704 (87.8%)	<0.0001	96 (39.8%)	233 (88.9%)	<0.0001
Yes	523 (59.0%)	127 (11.9%)		378 (58.6%)	98 (12.2%)		145 (60.2%)	29 (11.1%)	
**ypN stage**									
ypN1	–	523 (49.1%)	–	–	383 (47.8%)	–	–	140 (53.4%)	–
ypN2	–	352 (33.1%)	–	–	268 (33.4%)	–	–	84 (32.1%)	–
ypN3	–	189 (17.8%)	–	–	151 (18.8%)	–	–	38 (14.5%)	–

Abbreviations: pCR = Partial clinical response; ER = Estrogen receptor; FEC = Fluorouracil, epirubicin hydrochloride, and cyclophosphamide; NAC = Neoadjuvant chemotherapy; cCR = Complete clinical response; SLN = Sentinel lymph node.

a Assessed on core biopsy before neoadjuvant chemotherapy.

#### 3.1.1 Independent predictive factors of axillary pCR vs. residual nodal disease

After multivariate analysis, independent predictive factors associated to nodal pCR were axillary cCR evaluated on imaging after NAC (OR 2.95, 95%CI 2.36–3.68, p < 0.0001), ER-/HER2+ (OR 3.34, 95%CI 2.02–5.52, p < 0.0001) or ER+/HER2+ (OR 2.40, 95%CI 1.58–3.65, p < 0.0001) or ER-/HER2- (OR 1.94, 95%CI 1.34–2.81, p = 0.0004) biomolecular subtypes, breast cCR (OR 2.63, 95%CI 2.06–3.37, p < 0.0001), Ki67 > 14% (OR 1.76, 95%CI 1.24–2.51, p = 0.001), and histological tumor type “Others” (OR 2.06, 95%CI 1.22–3.47, p = 0.007), as showed in Table 2.

**Table 2:** Multivariate analysis for prediction of axillary pCR vs. residual nodal disease.

	ypN0 vs. ypN+ (computed the probability of ypN0)		
	OR	95%CI	P Value
**Age at diagnosis**	0.99	0.98–1	0.21
**Pre-treatment clinical T stage**			
cT2	1.19	0.85–1.66	0.31
cT3	1.14	0.76–1.71	0.52
cT4	0.99	0.66–1.48	0.96
cT1	–	–	–
**Histological type**^a^			
Invasive ductal	1.37	0.86–2.17	0.18
Others	2.06	1.22–3.47	0.007
Invasive lobular	–	–	–
**Grading**^a^			
G3	1.09	0.87–1.36	0.48
G1-2	–	–	–
**Biomolecular subtype**^a^			
ER+/HER2+	2.40	1.58–3.65	<0.0001
ER-/HER2+	3.34	2.02–5.52	<0.0001
ER-/HER2-	1.94	1.34–2.81	0.0004
ER+/HER2-	–	–	–
**Ki67 index**^a^			
>14%	1.76	1.24–2.51	0.001
≤14%	–	–	–
**Progesterone receptor**^a^			
Negative	1.35	1.00–1.82	0.051
Positive	–	–	–
**NAC regimen**			
Type 2	0.98	0.68–1.40	0.91
Type 3	1.14	0.70–1.83	0.60
Type 4	0.79	0.51–1.23	0.30
Type 1	–	–	–
**Post-NAC clinical axillary status**			
Negative	2.95	2.36–3.68	<0.0001
Positive	–	–	–
**Post-NAC breast cCR**			
Yes	2.63	2.06–3.37	<0.0001
No	–	–	–

Abbreviations: pCR = Partial clinical response; ER = Estrogen receptor; NAC = Neoadjuvant chemotherapy; cCR = Complete clinical response.

a Assessed on core biopsy before neoadjuvant chemotherapy.

#### 3.1.2 Development and internal validation of a nomogram to predict axillary pCR after neoadjuvant chemotherapy

**Fig. 1:**
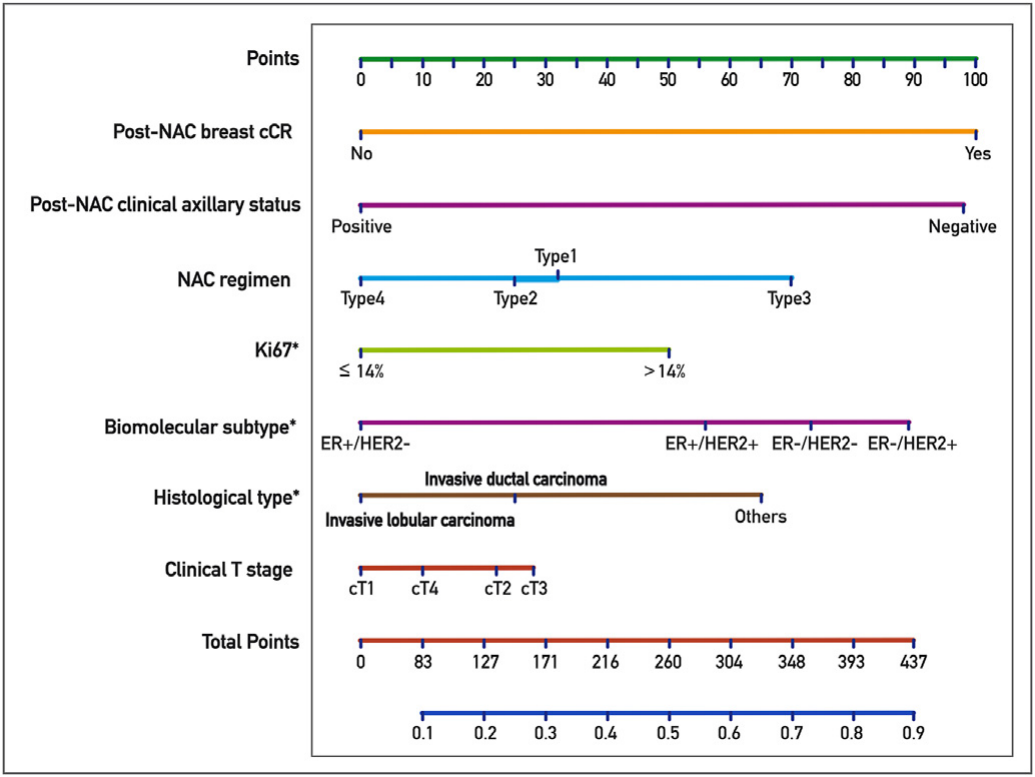
Nomogram to predict the individual probability of nodal pCR after NAC. ∗Assessed on core biopsy before neoadjuvant chemotherapy.

**Fig. 2:**
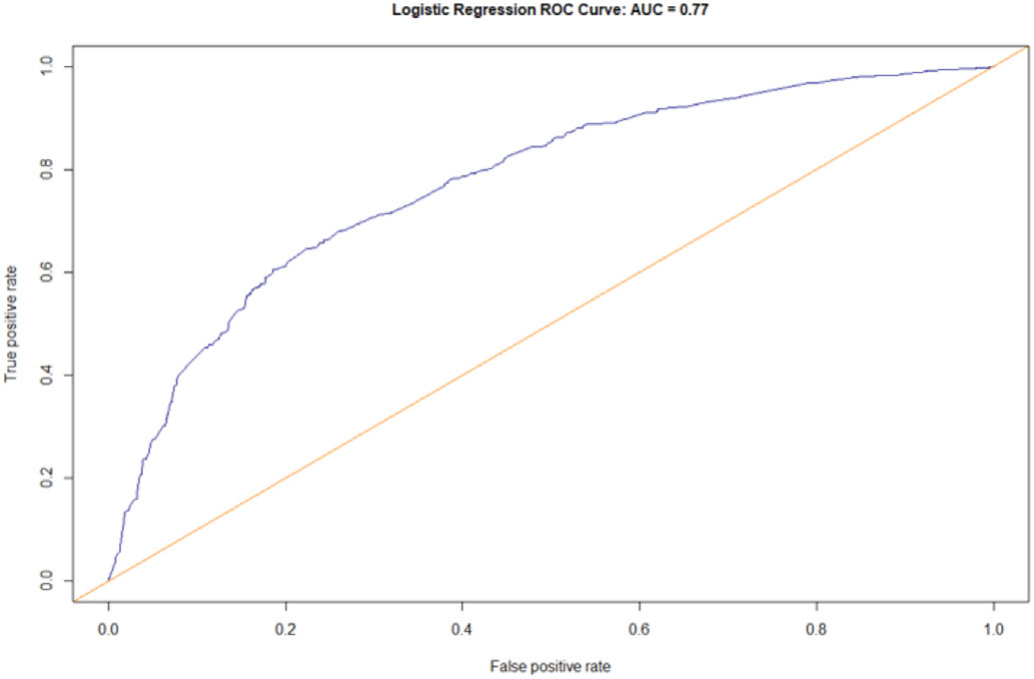
Performance of the proposed nomogram in predicting the individual probability of nodal pCR after NAC evaluated by ROC curve.

The nomogram was constructed starting from the higher ß coefficient observed in multivariate analysis, associated to the presence of breast cCR after NAC (β = 1.09, as shown in Table 3tbl3): this was considered the driver variable for development of the nomogram. Based on their ß coefficients, axillary cCR after NAC evaluated on imaging (β = 1.07) was matched with a score of 98, ER-/HER2-breast cancer (β = 0.80) with a score of 73, ER-/HER2+ subtype (β = 0.97) with a score of 89, ER+/HER2+ subtype (β = 0.61) with a score of 56, Ki67 > 14% at CB (β = 0.55) was matched with a score of 50, NACT's Type3 (β = 0.76) was matched with a score of 70, and histological tumor type at core biopsy “Others” (β = 0.71) with a score of 65. After accounting for the independent predictive factors for axillary pCR, pre-treatment clinical T stage was considered to be relevant although not statistically significant and was included in the model (Fig. 1; Table 3): its presence in the model has improved AIC value. A ROC curve based on predicted probability of axillary pCR was designed (Fig. 2). The nomogram showed a sensitivity of 71% and a specificity of 73% for axillary pCR. AUC was equal to 0.77 (95%CI [0.75–0.80]). Then, an internal validation of model accuracy was performed by bootstrap technique. The optimism index was equal to 0.01, and the corrected AUC after bootstrap was 0.76.

**Table 3:** Nomogram to predict the individual risk of nodal pCR after NAC.

	Model for nomogram (computed the probability of ypN0)			
	β coefficient	95%CI	P Value	Score
**Pre-treatment clinical T stage**				
cT2	0.24	−0.14-0.63	0.21	22
cT3	0.31	−0.16-0.78	0.19	28
cT4	0.11	−0.36-0.57	0.65	10
cT1	–	–	–	0
**Histological type**^a^				
Invasive ductal	0.27	−0.26-0.81	0.32	25
Others	0.71	0.09–1.32	0.02	65
Invasive lobular	–	–	–	0
**Biomolecular subtype**^a^				
ER+/HER2+	0.61	0.12–1.11	0.02	56
ER-/HER2+	0.97	0.44–1.51	0.003	89
ER-/HER2-	0.80	0.46–1.14	<0.0001	73
ER+/HER2-	–	–	–	0
**Ki67 index**^a^				
>14%	0.55	0.16–1.93	0.005	50
≤14%	–	–	–	0
**NAC regimen**				
Type 1	0.34	−0.24-0.93	0.25	32
Type 2	0.28	−0.25-0.81	0.31	25
Type 3	0.76	0.17–1.35	0.01	70
Type 4	–	–	–	0
**Post-NAC clinical axillary status**				
Negative	1.07	0.80–1.34	<0.0001	98
Positive	–	–	–	0
**Post-NAC breast cCR**				
Yes	1.09	0.81–1.38	<0.0001	100
No	–	–	–	0

Abbreviations: pCR = Partial clinical response; NAC = Neoadjuvant chemotherapy; ER = Estrogen receptor; cCR = Complete clinical response.

a Assessed on core biopsy before neoadjuvant chemotherapy.

#### 3.1.3 External validation of the nomogram to predict axillary pCR after neoadjuvant chemotherapy

External validation is essential to support generalizability of the prediction nomogram for patients other than those in the development cohort [26]. The same model developed in Table 3 was applied on the external validation cohort: the accuracy of the nomogram was confirmed with an AUC of 0.77 (95%CI [0.73–0.82]). Calibration on external cohort was performed by graphical method (Supplementary Fig S3).

## 4 Discussion

A tout-court omission of ALND in patients with residual disease in SLN after NAC could be oncologically unsafe, as evidences are still poor. The biological meaning of even minimal nodal disease is unclear after NAC, and axillary tumor burden beyond SLN is currently not estimable. Recently, a high frequency of non-SLN metastases was reported in patients with involved SLN after NAC, exceeding 40% [27]. Conversely, in the present series 1–3 involved nodes only were found in 49.1% of patients. Therefore, in about half of patients no further axillary disease would be found on ALND after SLNs biopsy. After publication of SENTINA, ACOSOG Z1071 and GANEA 2 trials [12,28,29], other trials have evaluated the feasibility of SLN biopsy after NAC [[30], [31], [32]]. But the false negative rate is still considered unacceptably high (11.9%–14.2%) and ALND is still largely performed, with significant sequelae in up to 60.3% of patients [29]. Alternative techniques such as TAD have been proposed, but more choices mean more questions: which lymph nodes should be clipped? Which is the best timing to perform nodal clipping? Furthermore, clipping several nodes could be risky and unfeasible, and radioactive seed is not widely available [18]. Therefore, a nomogram to reliably predict axillary pCR might be useful in selecting those patients who may avoid upfront ALND in favor of SLN biopsy, but also to guide the type of axillary surgery in case of minimal residual disease in the SLNs.

In the present multicenter study a nomogram for prediction of axillary pCR in initially node-positive breast cancer has been developed, with a sensitivity of 71% and a specificity of 73% (AUC 0.77), confirmed after external validation. Expectedly, the strongest predictors were clinical N stage after NAC (OR 2.95, p < 0.0001), ER-/HER2+ disease (OR 3.34, p < 0.0001) and presence of breast cCR (OR 2.63, p < 0.0001). Interestingly, the present study evidenced the value of ultrasound to re-stage the axilla after NAC. Indeed, 67.6% of ypN + patients had a positive axilla at pre-operative re-staging. A correlation between clinical axillary re-staging after NAC and the presence of residual nodal disease has been previously reported, but with a lower accuracy. Indeed, a secondary analysis of the ACOSOG Z1071 trial found that up to 56.5% of patients with a clinically negative axilla had residual nodal disease [33].

Another predictor of axillary pCR is breast cCR (OR 2.48, p < 0.0001), since it was observed after neoadjuvant treatment in 45.0% of axillary pCR patients vs. 15.3% only in the residual nodal disease group (p < 0.0001). Notably, clinical but not pathological response was considered for development of the predictive nomogram, since breast pCR it is assessable only after surgery. The relation between breast and axillary pCR is already established, and patients who achieve breast pCR are also node-negative on final pathology in up to 59.0% of cases. Due to the high probability of axillary pCR in this subset of patients, breast pCR itself has been recently suggested to be a main driver for possible omission of any axillary surgery after NAC [33,34]. Furthermore, in patients with combined breast and axillary pCR survival is particularly high, up to 94% at 5 years, and it is mainly driven by response to NAC than initial nodal status [35].

The above-mentioned predictors are evaluated only at the end of neoadjuvant treatment. Conversely, biomolecular subtype, grading and Ki67 were strong predictors provided before starting of NAC, thus independent from clinical evaluation of response. These variables *a priori* predicted axillary pCR, which was highly correlated with ER-/HER2- (OR 1.89, p = 0.001), ER+/HER2+ (OR 2.26, p = 0.0002) and ER-/HER2+ (OR 3.26, p < 0.0001) breast cancers. ER+/HER2-cancers were less likely to achieve axillary pCR, representing up to 60.2% of patients with residual nodal disease. A large study recently reported a pCR rate of 0.3% for Luminal A cancers vs. 38.7% in HER2-positive cases, and molecular subtype independently predicted both pCR and overall survival [36]. In the present study, a Ki67 < 14% was associated with lower probability of nodal pCR (OR 0.52, p = 0.0005). The value of Ki67 has been suggested in some studies and a meta-analysis confirmed that a high baseline Ki67 predicts a higher probability of pCR irrespectively other variables, even in ER + breast cancer [37]. The reason probably is that highly proliferating malignancies are more susceptible to chemotherapy, and Ki67 is a marker of cell proliferation [38]. However, optimal cut-off for Ki67 still needs to be determined in the neoadjuvant setting [39].

A large retrospective study on more than 13,000 patients from the National Cancer Data Base (NCDB) has developed a nomogram with an AUC of 0.77, similar to our findings [40]. However, data regarding clinical tumor size or clinical axillary status after NAC are not reported in the NCDB: the former has been surrogated by pathological tumor size, and the latter has neither been cited, despite post-NAC clinical axillary status is of paramount importance in prediction of nodal pCR. Other 2 nomograms have been developed at the MD Anderson Cancer Center (Texas, USA) and externally validated at the European Institute of Oncology (Italy) but: 1) again, post-NAC clinical axillary status was not considered; 2): breast cCR/pCR was not included in the model and 3) isolated tumor cells were considered as positive nodes [41]. Also a Dutch study reported a nomogram, but a very low sensitivity (43%) limited its use for individual decisions. Furthermore, in the Dutch study only 54.2% of HER2+ patients received trastuzumab, suggesting a non-contemporary clinical practice [42].

## 5 Conclusions

The strategy for axillary surgery after NAC is still greatly debated. National guidelines and scientific society recommendations still do not provide clarity, as strong evidences are lacking. Prospective trials are ongoing, assessing survival outcomes and quality of life after different strategies in patients who convert from cN + to ycN0. But once ascertained the most suitable surgery, the question is which patients will be safely treated by SLN biopsy or TAD and which ones will reasonably require ALND. The predictive nomogram-based online tool developed in our study could identify patients eligible to be treated by mini-invasive axillary surgery after NAC. Not only this tool could help to tailor axillary surgery, but it could also be used to reduce the need for ALND also in patients with residual nodal disease in the SLN, if the likelihood of a positive axilla after NAC is low.

## Funding

This research did not receive any specific grant from funding agencies in the public, commercial, or not-for-profit sectors.

## Declaration of competing interest

None.
