# Supplementary material for: Development of a novel nomogram-based online tool to predict axillary status after neoadjuvant chemotherapy in cN+ breast cancer: A multicentre study on 1,950 patients
Source: Breast. 2021 Oct 2;60:131–7. doi: 10.1016/j.breast.2021.09.013 (PMC8503563; doi:10.1016/j.breast.2021.09.013)
Supplement: Multimedia component 1 [file mmc1.docx]

**Development of a novel nomogram-based online tool to predict axillary status after neoadjuvant chemotherapy in cN+ breast cancer: a multicentre study on 1,950 patients**

**Supplementary Methods**

*Nomogram development*

Baseline pre-neoadjuvant chemotherapy (NAC) and post-NAC variables were collected for each patient. A multivariate analysis with a binomial logistic model was performed, and all relevant variables possibly related to nodal partial clinical response (pCR) were included. Based on the Akaike Information Criterion (AIC), seven variables were selected for the nomogram (a lower AIC value indicated a better model fit). To develop the nomogram, the variable with the higher model’s ß coefficient was chosen as the driver variable on which the predictive model was based, independently from its statistical significance. A score of 0 points was attributed to the reference value of that variable, and 100 points to the other category’s value; any intermediate values were matched with corresponding points. Then, each other variable was matched with a score based on the ratio between its ß coefficient and ß coefficient of the driver variable, independently from its statistical significance too:

$$\mathrm{Score}_{\mathrm{variable}}=\frac{\beta_{\mathrm{variable}}}{\beta_{\mathrm{driver}}}*100$$

Calibration was performed by graphical method (Supplementary Fig S1). The nomogram was then applied to each patient to calculate the individual risk of nodal pCR after NAC. A Receiver Operating Characteristic (ROC) curve with relative Area Under Curve (AUC) was designed to assess accuracy, sensitivity and specificity, and the overall performance measure given by explained variance (coefficient of determination R^2^) of the model using the definition proposed by Nagelkerke was assessed (R^2^=0.3, p <0.0001). Furthermore, the fit’s linear regression of the predicted values relative to the developed nomogram was evaluated (Supplementary Fig S2). An internal validation was performed with bootstrap method: the original patient population was re-sampled 500 times and the optimism index (the mean of differences between AUC on bootstrap sample and AUC on original sample) was calculated. Optimism is the amount by which the AUC (or “the apparent prediction accuracy”) overestimates the true prediction accuracy of the model. Then, the corrected AUC after bootstrap was reported.

Finally, the developed model was validated in the external validation cohort in order to verify the applicability of the nomogram on a different and independent cohort. The relative AUC of the model in this subset was assessed. Also in this context, calibration was performed by graphical method (Supplementary Fig S3).

*Statistical analysis*

Categorical variables were compared using χ2 test, while continuous variables were compared using Student’s T test or non-parametric Wilcoxon test in case of non-normal distribution of the variable. A multivariate analysis with a binomial logistic model was performed and the Akaike Information Criterion (AIC) was evaluated (a lower AIC value indicated a better model fit) to select the appropriate number of variables for nomogram. A Receiver Operating Characteristic (ROC) curve with relative Area Under Curve (AUC) was designed to assess accuracy, sensitivity and specificity, and the overall performance measure given by explained variance (coefficient of determination R^2^) of the model using the definition proposed by Nagelkerke was assessed.

**Supplementary figures**


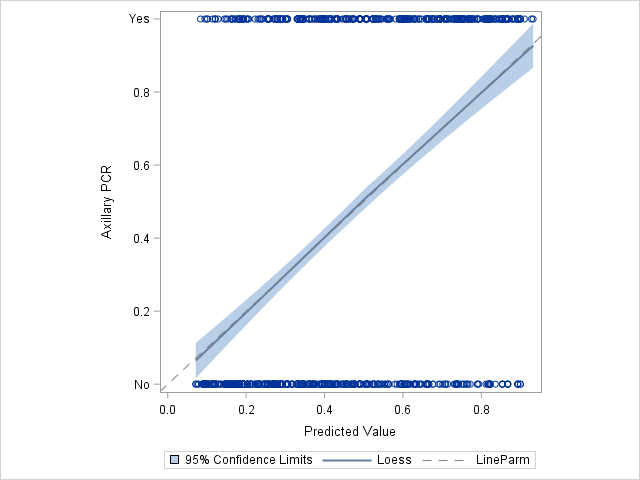


**Supplementary Fig. S1** Calibration model for the developed nomogram


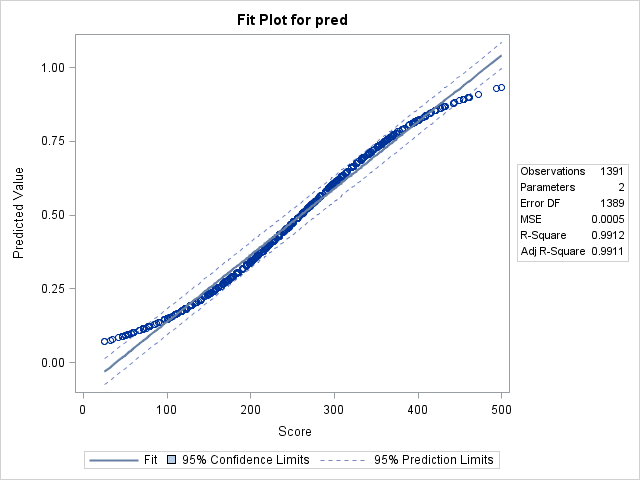


**Supplementary Fig. S2** Fitting plot for prediction related to the developed nomogram model


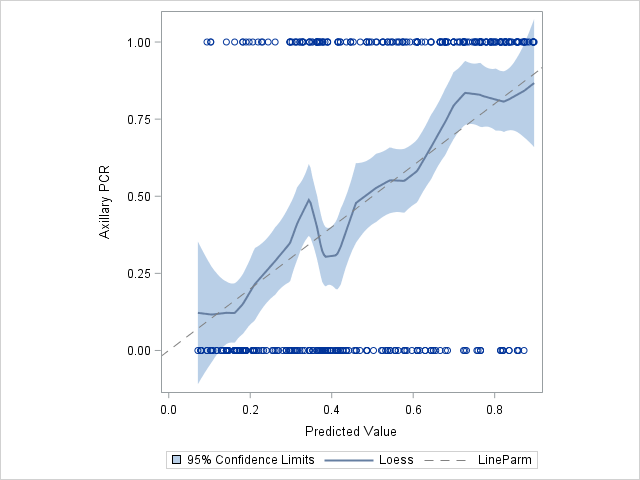


**Supplementary Fig. S3** Calibration model for the external validation of nomogram
